# Supplementary material for: The avian fossil record in Insular Southeast Asia and its implications for avian biogeography and palaeoecology
Source: PeerJ. 2014 Mar 11;2:e295. doi: 10.7717/peerj.295 (PMC3961167; doi:10.7717/peerj.295)
Supplement: Table S1 — † indicates an extinct species; (†) indicates a possibly extinct species; * indicates a species that is no longer present in Insular Southeast Asia. [file peerj-02-295-s001.pdf]

LOCALITY AND AGE		Family	Species		REFERENCE
**Sumatra**	**Sipang, Sangkarewang Formation, ?Eocene**				Lambrecht, 1931
		Fregatidae/Suloi	*Protoplotus beauforti*†		
	**Kandi, Sawahlunto Formation, Oligocene**				Zaim et al., 2011
			Aquatilavipes wallacei		
			Aquatilavipes sp. A		
**Java**	**Trinil, Middle Pleistocene**				Weesie, 1982
		Anatidae	*Tadorna tadornoides*	Australian shelduck	
			Branta cf. *ruficollis**	red-breasted goose	
		Ciconiidae	*Leptoptilos* cf. *dubius**	greater adjutant	
			Ephippiorhnchus cf. *asiaticus*	black-necked stork	
	**Sumber Kepuh, Middle Pleistocene**				Weesie, 1982
		Phasianidae	*Pavo muticus muticus*	grean peafowl	
	**Watoealang, Late Pleistocene**				Wetmore, 1940
		Ciconiidae	*Leptoptilos titan*†	giant stork	
		Gruidae	*Grus grus**	common crane	
		Accipitridae	Aegypinae?	vulture	
	**Wajak, Late Pleistocene**				van den Brink, 1982
		Strigiformes	indet	owl	
		Passeriformes	indet	songbird	
**Borneo**	**Great Cave of Niah, (Sarawak), Late Pleistocene - Holocene**				Stimpson, 2009; 2010; 2013
		Accipitridae	*Macheiramphus alcinus*	bathawk	
			Haliastar indus	Brahminy kite	
			Accipiter cf. *trivirgatus*	crested goshawk	
			Ictinaetus malayensis	Indian black eagle	
			Spizaetus sp.	hawk eagle	
			Butastur indicus	grey-faced buzzard	
			Spilornis cheela	crested serpent eagle	
		Tytonidae	*Phodilus badius*	oriental bay owl	
		Strigidae	*Bubo ketupu*	buffy fish owl	
			Bubu sumatranus	barred eagle owl	
			Strix leptogrammica	brown wood owl	
		Apodidae	*Collocalia esculenta*	glossy swiftlet	
			Aerodramus salangana	mossy-nest swiftlet	
			Aerodramus maximus	black-nest swiftlet	
		Bucerotidae	*Anorrhinus galeritus*	bushy crested hornbill	
			Anthracoceros albirostiris	oriental pied hornbill	
			Anthracoceros malayanus	black hornbill	
			Anthracoceros sp.	hornbill	
			Aceros corrugatus	wrinkled hornbill	
			Rhyticeros undulatus	wreathed hornbill	
			Buceros sp.	hornbill	
		Phasianidae	*Lophura ignita*	crested fireback	
			Lophura erythrophthalma	crestless fireback	
			inter. *Lophura* sp.	gallopheasant	
			Arborophila sp.	partridge	
		Estrildidae	*Lonchura* cf. *fuscans*	dusky munia	
		Corvidae	*Cissa chinensis*	green magpie	
			Cissa sp.	magpie	
**Flores**	**Liang Bua, Late Pleistocene - Holocene**				Meijer et al., 2013
		Anatidae	Anatidae gen. et sp. indet	ducks and allies	
		Ciconiidae	*Leptoptilos robustus*†	giant stork	
		Accipitridae	*Haliastur* cf. *indus*	Brahminy Kite	
			Trigonoceps sp.*	white-headed vulture	
			Accipiter sp.	goshawk	
			Aquila sp.	eagle	
		Rallidae	*Gallirallus* sp.	rail	
			Porzana sp.	crake	
		Charadiidae	*Pluvialis fulva*	Pacific golden plover	
		Scolopacidae	*Gallinago* sp.	snipe	
			Actitis hypoleucos	common sandpiper	
		Turnicidae	*Turnix* sp.	buttonquail	
		Columbidae	Columbidae gen. et sp. indet	pigeon	
			Streptopelia cf. *bitorquata*	island collared dove	
			aff. *Macropygia/Ptilinopus*	dove	
			Macropygia sp.	cuckoo dove	
			Ptilinopus sp.	fruit dove	
			Ducula sp.	imperial pigeon	
		Psittacidae	*Geoffroyus* cf. *geoffroyi*	red-cheeked parrot	
		Tytonidae	*Tyto* sp.(†)	barn owl	
		Strigidae	Strigidae gen. et sp. indet	owl	
			Otus sp.	scops owl	
		Apodidae	*Collocalia esculenta*	glossy swiftlet	
			Aerodramus cf. *fuciphagus*	edible-nest swiftlet	
		Alcedinidae	Halcyoninae gen. et sp. indet	kingfisher	
			Halcyon sp.	tree kingfisher	
**Palawan**	**Cave sites, Terminal Pleistocene - Holocene**				Reiss & Garong, 2001
		Strigidae	*Otus* sp.	scops owl	
		Apodidae	*Aerodramus* cf. *salanganus*	mossy-nest swiftlet	
			Collocalia cf. *esculenta*	glossy swiftlet	
			Collocalia cf. *troglodytes*	pygmy swiftlet	
		Timaliidae	Timaliidae sp.	Old World babblers	
		Turdidae	*Zoothera dauma*	scaly trush	
		Sylviidae	Sylviidae sp.	Old World warblers	
		Monarchidae	*Hypothymis azurea*	black-naped monarch	
		Motacillidae	*Motacilla* sp.	wagtail	
		Laniidae	*Lanius cristatus*	brown shrike	
		Dicaeidae	*Dicaeum* cf. *aeruginosum*	striped flowerpecker	
		Zosteropidae	*Zosterops* cf. *montanus*	mountain white-eye	
